# Absence of p55 TNF Receptor Reduces Atherosclerosis, but Has No Major Effect on Angiotensin II Induced Aneurysms in LDL Receptor Deficient Mice

**DOI:** 10.1371/journal.pone.0006113

**Published:** 2009-07-07

**Authors:** Sofia Xanthoulea, Melanie Thelen, Chantal Pöttgens, Marion J. J. Gijbels, Esther Lutgens, Menno P. J. de Winther

**Affiliations:** 1 Department of Molecular Genetics, Cardiovascular Research Institute Maastricht, Maastricht University, Maastricht, The Netherlands; 2 Department of Pathology, Cardiovascular Research Institute Maastricht, Maastricht University, Maastricht, The Netherlands; University of Sheffield, United Kingdom

## Abstract

**Background:**

The aim of the current study was to investigate the role of p55 TNF Receptor (p55 TNFR), the main signaling receptor for the pro-inflammatory cytokine tumor necrosis factor (TNF), in the development of two vascular disorders: atherosclerosis and angiotensin (Ang) II-induced abdominal aortic aneurysms (AAA).

**Methodology/Principal Findings:**

p55 TNFR deficient mice were crossed to an LDL receptor deficient background and were induced for the development of either atherosclerosis or AngII-induced AAA, and compared to littermate controls, wild-type for p55 TNFR expression. p55 TNFR deficient mice developed 43% smaller atherosclerotic lesions in the aortic sinuses compared to controls. Moreover, expression of CD68, a macrophage specific marker, exhibited a 50% reduction in the aortic arches. Decreased atherosclerosis correlated with a strong down-regulation in the expression of adhesion molecules, such as VCAM-1 and ICAM-1, by p55 TNFR deficient endothelium. In addition, expression levels of the pro-inflammatory cytokines and chemokines TNF, IL-6, MCP-1 and RANTES were significantly reduced in aortas of p55 TNFR deficient mice. In contrast, in the AngII-induced model of AAA, p55 TNFR deficiency correlated with a slight trend towards increased aneurismal lethality, but the incidence of aortic rupture due to a dissecting aneurysm, and the expansion of the suprarenal aorta were not significantly different compared to controls.

**Conclusion/Significance:**

We found that p55 TNFR expression promotes atherosclerosis, among other mechanisms, by enhancing expression of endothelial adhesion molecules, while it seems to have no major role in the development of AngII-induced AAA.

## Introduction

Vascular inflammatory processes, characterized by the accumulation in the arterial wall of immune cells like monocytes/macrophages and lymphocytes, are crucial events in the pathogenesis of major vascular diseases such as atherosclerosis and abdominal aortic aneurysms (AAA) [1], [2]. Tumor necrosis factor (TNF) is a major cytokine with a pivotal role in orchestrating inflammatory responses and p55 TNFR mediates the majority of TNF responses [3].

In atherosclerosis, TNF is generally considered to promote plaque growth and progression since disease induction in TNF deficient mice resulted in reduced development of atherosclerotic lesions as well as their reduced progression towards more advanced stages [4], [5], [6], [7]. The role of the p55 TNFR in atherogenesis is however less established. Total-body p55 TNFR deficiency was shown to either have no effect on lesion size, composition or features of plaque destabilization in very advanced atherosclerosis [8], or to result in bigger lesions in C57BL/6 mice fed an atherogenic diet, suggesting an athero-protective function [9].

Regarding the cell-type specific role of this receptor, we have recently shown that bone-marrow derived p55 TNFR promotes atherosclerosis development by enhancing lesional foam-cell formation and by promoting the expression of pro-atherosclerotic chemokines, like MCP-1 [10]. In addition, by using a model of carotid artery-to-carotid artery interposition grafting in which p55 TNFR deficient (but ApoE sufficient) carotid arteries were grafted into ApoE deficient recipients, it was shown that arterial wall (i.e. smooth muscle and endothelial cell) p55 TNFR expression is pro-atherogenic [11]. However, as also indicated by the authors, this study presented some limitations since it appeared that there was a slight alloimmune reaction accelerating atherosclerosis, possibly elicited by ApoE secreted by graft smooth muscle cells. This might complicate the elucidation of the actual role of vascular wall p55 TNFR in atherogenesis. In addition, compared with the total-body p55 TNFR deficiency studies, these results suggest the possibility that p55 TNFR expression in different cell types might play opposing roles in disease development.

The role of the TNF-p55 TNFR signaling in the development of abdominal aortic aneurysms is not defined. Both serum [12] and aneurismal tissues [13] from AAA patients have increased TNF levels, implicating a role for this cytokine in disease pathogenesis. However, to our knowledge, mouse models of AAA have not been applied in either TNF or p55 TNFR deficient background.

In order to elucidate the role of p55 TNFR signaling in these two vascular disorders, we have studied disease development in total-body p55 TNFR deficient mice or their littermate wild-type controls in a hyperlipidemic LDL receptor deficient background, a well-established and physiologic model of these diseases. We show that p55 TNFR expression promotes atherosclerosis in two different sites prone to atherosclerotic lesion development like the aortic sinuses and the aortic arches. Concomitant to additional mechanisms identified previously [10], here we show that, in a physiological setting, the pro-atherosclerotic action of this important innate receptor is also mediated by inducing adhesion molecule expression in endothelial cells and by enhancing production of pro-atherosclerotic cytokines and chemokines at the arterial wall. However, in a hyperlipidemia-accelerated [15], [16], [17] AngII-induced model of AAA, the role of p55 TNFR signaling appears to be secondary. Although we observed a trend towards an increased aneurismal lethality in the p55 TNFR deficient mice, differences were not significant. Moreover, additional parameters of disease susceptibility like the incidence of dissecting aneurysms and the suprarenal aortic diameters were also not significantly affected by the absence of the receptor.

## Results

### p55 TNFR deficient mice develop smaller atherosclerotic plaques

**Figure 1:**
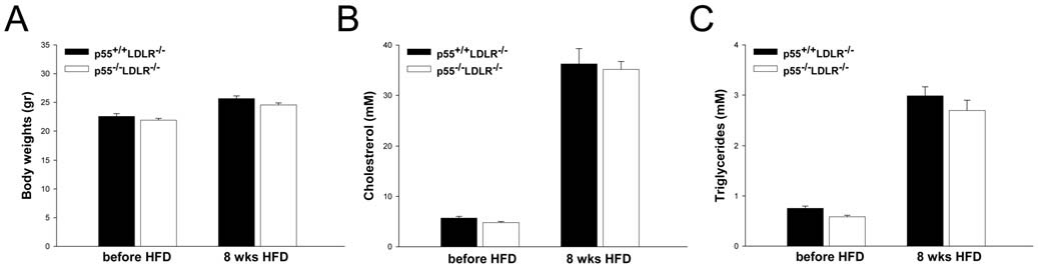
Body weight and plasma lipid levels. (A) Body weight (B) plasma cholesterol and (C) plasma triglyceride levels in p55^+/+^LDLR^−/−^ (n = 18) and p55^−/−^LDLR^−/−^ (n = 16) mice before and after 8 weeks of high fat feeding.

p55 TNFR deficient (p55^−/−^LDLR^−/−^; n = 16) and littermate control (p55^+/+^LDLR^−/−^; n = 18) mice were fed a high fat diet for 8 weeks and blood samples were collected at the beginning and at the end of the feeding period. Plasma cholesterol and triglyceride levels increased upon high fat diet but no significant differences between the groups were observed. Body weights were also comparable (Fig. 1A, B, C).

**Figure 2:**
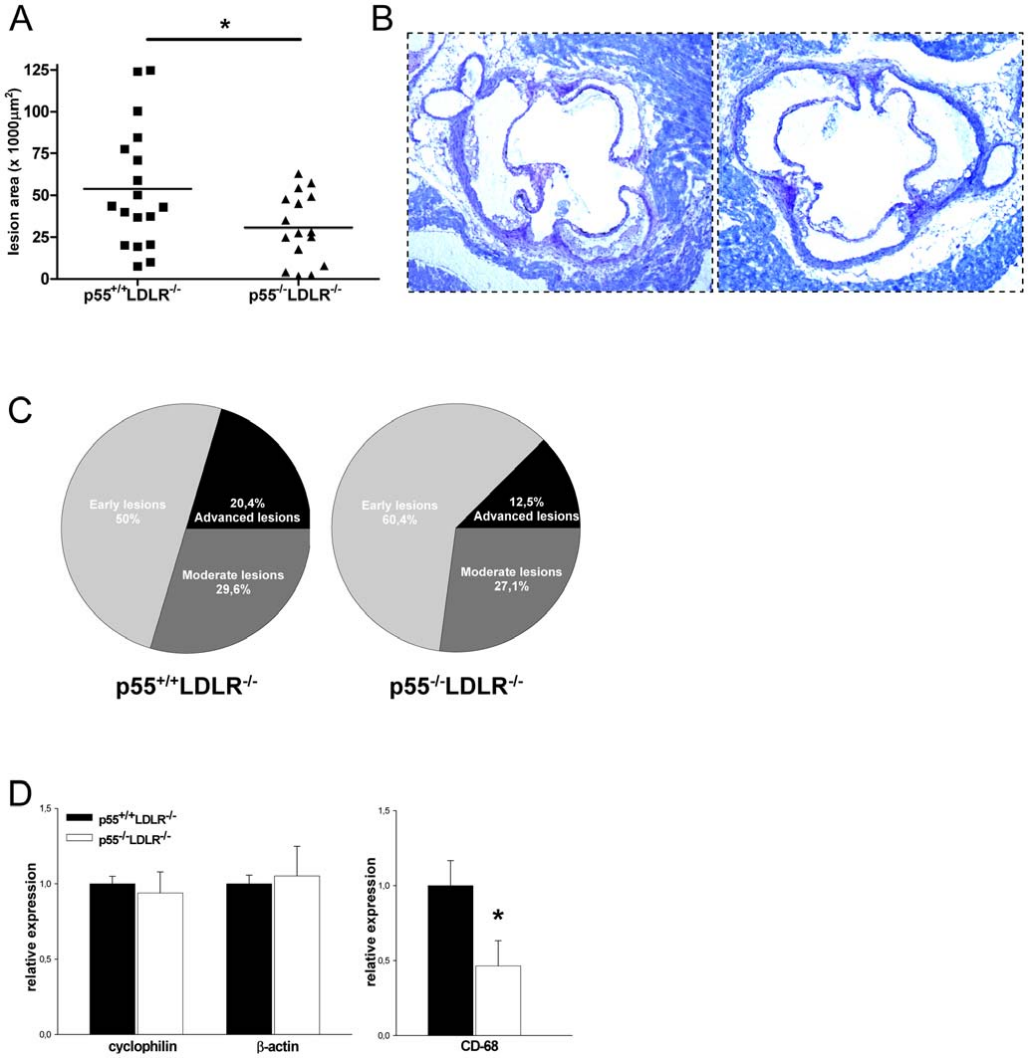
Atherosclerosis quantification. (A) Atherosclerotic lesion area in the aortic sinuses of p55^+/+^LDLR^−/−^ (squares, n = 18) and p55^−/−^LDLR^−/−^ (triangles, n = 16) mice. Each symbol represents one animal; bars represent means. * p = 0.02 by Student's t-test. (B) Representative lesions from p55^+/+^LDLR^−/−^ and p55^−/−^LDLR^−/−^ mice are shown. Original magnification×40. (C) Lesion classification according to severity. (D) Gene expression analysis in p55^+/+^LDLR^−/−^ (n = 10) and p55^−/−^LDLR^−/−^ (n = 8) aortic arches. Values are represented relative to expression in p55^+/+^LDLR^−/−^ arches. * p = 0.03 by Student's t-test. Error bars indicate SEM.

At sacrifice, the hearts and the aortic arches were isolated. Assessment of atherosclerotic lesion size in the aortic sinus area showed that p55^−/−^LDLR^−/−^ mice developed 43% smaller atherosclerotic plaques compared to p55^+/+^LDLR^−/−^ littermates (Fig. 2A, B; p = 0.02). In addition, lesions were categorized for severity with a scale from 1–3: (1) early lesions with fatty streaks containing only macrophage derived foam-cells, (2) moderate lesions characterized by the additional presence of a collagenous cap, (3) advanced lesions with involvement of the media and increased collagen content. p55^+/+^LDLR^−/−^ mice had 50% early lesions, 29.6% moderate and 20.4% advanced lesions while these percentages were 60.4%, 27.1% and 12.5% respectively for the p55^−/−^LDLR^−/−^ mice (Fig. 2C). Thus, p55 TNFR ablation inhibits atherosclerosis development.

Next, RNA was isolated from aortic arches of p55^−/−^LDLR^−/−^ and control mice and expression levels of the macrophage marker CD-68 were determined. Expression levels of the housekeeping genes cyclophilin and β-actin, used as controls, were similar between the groups. Aortic arches of p55^−/−^LDLR^−/−^ mice showed approximately 50% less CD-68 macrophage-marker expression compared to controls, suggesting reduced presence of macrophages and thereby smaller plaques (Fig. 2D). Hence, p55 TNFR promotes the accumulation of macrophages and the development of atherosclerotic lesions in two different atherosclerosis prone sites as the aortic valve area and the aortic arches.

### Reduced expression of adhesion molecules by p55 TNFR deficient endothelium

**Figure 3:**
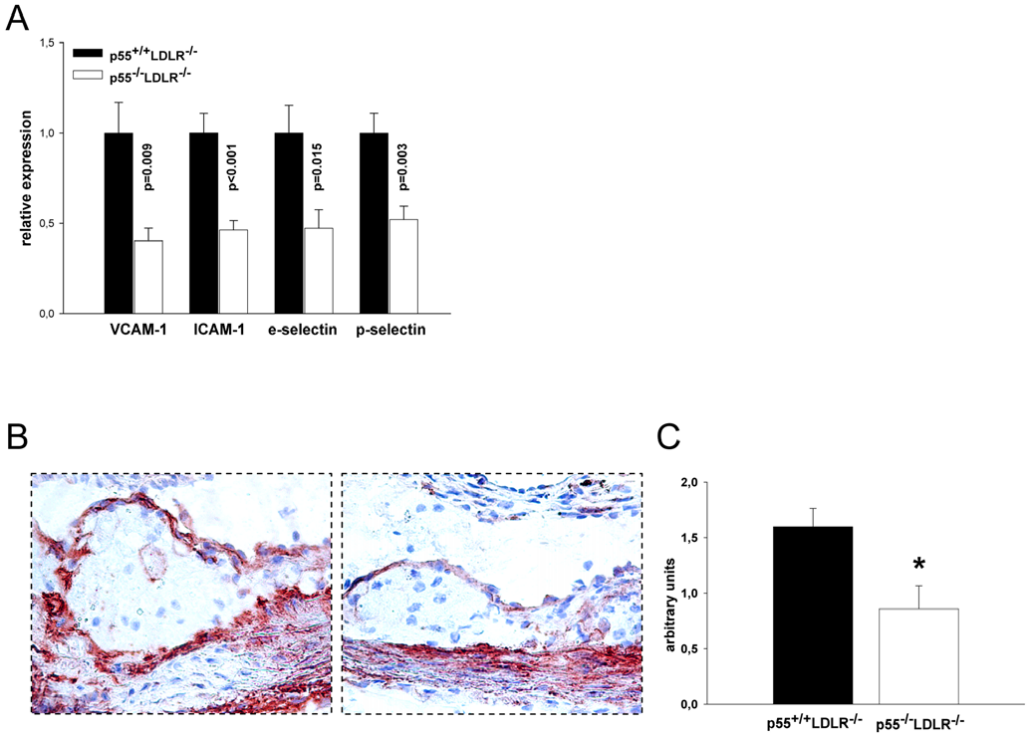
Adhesion molecules expression analysis. (A) Relative mRNA levels of VCAM-1, ICAM-1, e-selectin and p-selectin in aortic arches from p55^+/+^LDLR^−/−^ (n = 10) and p55^−/−^LDLR^−/−^ (n = 8) mice. Values are represented relative to expression in p55^+/+^LDLR^−/−^ arches. (B) Immunohistochemical staining of VCAM-1 expression on sections from aortic valve areas indicating a less intense endothelial staining in p55^−/−^LDLR^−/−^ mice. Original magnification×200. (C) Staining quantification. * p = 0.01 by Student's t-test. Error bars indicate SEM.

Expression of adhesion molecules by the vascular endothelium is thought to be one of the first events promoting atherogenesis, and p55 TNFR has a crucial role in the TNF-induced expression of adhesion molecules and consequent leukocyte organ infiltration [18], [19]. We thus examined by quantitative real-time PCR (qRT-PCR) the expression levels of four adhesion molecules, VCAM-1, ICAM-1, e-selectin and p-selectin, that are important for the development of atherosclerosis [20] on RNA isolated from the aortic arches of p55^−/−^LDLR^−/−^ and control mice. mRNA levels of all these adhesion molecules were significantly and approximately 50–60% reduced in aortas of p55^−/−^LDLR^−/−^ mice compared to controls (Fig. 3A). In addition, atherosclerotic lesions were immunostained with an anti-VCAM-1 specific antibody and the intensity of endothelial VCAM-1 staining was semi-quantitatively determined by two independent observers. This confirmed the reduced expression of VCAM-1 by p55 TNFR deficient endothelium in p55^−/−^LDLR^−/−^ lesions (Fig. 3B, C). These results are in line with previous findings regarding the crucial role of p55 TNFR in the control of adhesion molecules expression by endothelial cells at inflammatory sites [11], [18], [19], and extend this pivotal function also to sites of developing atherosclerotic lesions in a physiological hyperlipidemia induced model of the disease.

### Reduced expression of pro-atherosclerotic cytokines and chemokines in aortas of p55 TNFR deficient mice

**Figure 4:**
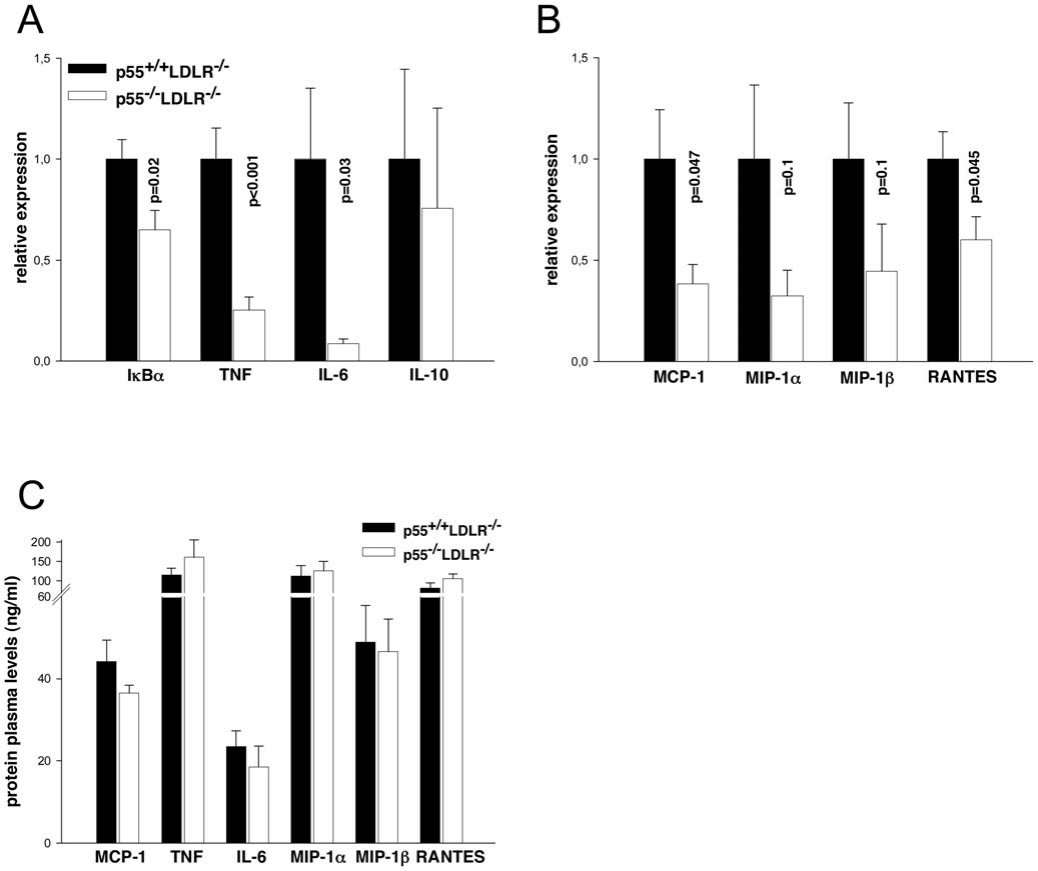
Cytokine and chemokine expression analysis. (A) Relative mRNA levels of IκBα, TNF, IL-6, IL-10 and (B) MCP-1, MIP-1α, MIP-1β, RANTES in aortic arches from p55^+/+^LDLR^−/−^ (n = 10) and p55^−/−^LDLR^−/−^ (n = 8) mice. Values are represented relative to expression in p55^+/+^LDLR^−/−^ arches. (C) Plasma levels of pro-inflammatory cytokines and chemokines (n = 12–15 mice/group) after 8 weeks of high fat feeding. Error bars indicate SEM.

Secretion of cytokines and chemokines in atherosclerotic plaques perpetuates the local inflammatory reaction and contributes to the further growth and development of the lesions [1]. p55 TNFR signaling induces the activation of transcription factors, like NF-κB, that regulate the expression of many pro-inflammatory and pro-atherosclerotic molecules [21]. We therefore examined by qRT-PCR the expression levels of a panel of cytokines and chemokines in aortic arches of p55^−/−^LDLR^−/−^ and control mice. Analysis of activation of the transcription factor NF-κB, as assessed by IκBα expression and by expression levels of the pro-inflammatory NF-κB target genes TNF and IL-6, showed that these were significantly reduced in p55^−/−^LDLR^−/−^ aortas (Fig. 4A). In contrast, the anti-inflammatory cytokine IL-10 was unaffected (Fig. 4A). In addition, expression levels of the pro-atherosclerotic chemokines MCP-1 and RANTES were also significantly lower in p55^−/−^LDLR^−/−^ aortas compared to controls (Fig. 4B). Expression levels of chemokines MIP-1α and MIP-1β were reduced, but differences did not reach statistical significance (Fig. 4B). Plasma concentrations of these inflammatory cytokines and chemokines, measured at the end of the experiment, indicated no significant differences between the groups (Fig. 4C). Thus, absence of p55 TNFR expression leads to a reduced expression of several pro-inflammatory and pro-atherosclerotic cytokines and chemokines at sites prone to atherosclerotic lesion development.

### General characterisation of AngII infused mice

**Figure 5:**
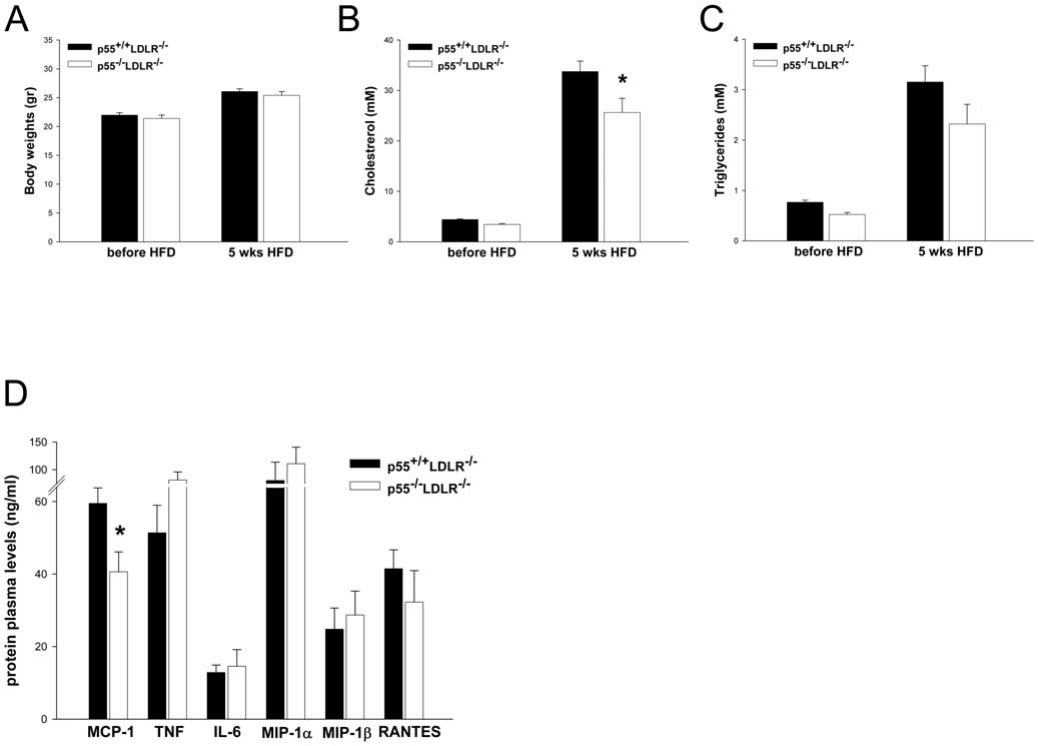
General characterization of AngII infused mice. (A) Body weight (B) plasma cholesterol and (C) plasma triglyceride levels in p55^+/+^LDLR^−/−^ and p55^−/−^LDLR^−/−^ mice before (n = 13–14 mice/group) and after 5 weeks of high fat feeding (4 weeks of AngII infusion; n = 8–12 mice/group). (D) Plasma levels of pro-inflammatory cytokines and chemokines (n = 7–9 mice/group) after 5 weeks of high fat feeding (4 weeks of AngII infusion). * p<0.05 by Student's t-test. Error bars indicate SEM.

To determine the involvement of p55 TNFR in the AngII-induced model of AAA formation, LDLR^−/−^ mice either wild-type (p55^+/+^LDLR^−/−^) or deficient for p55 TNFR (p55^−/−^LDLR^−/−^) were fed a fat-enriched diet and infused with either saline (n = 3/group) or AngII (1000 ng/kg/min; n = 13–14/group) for 28 days. No differences in body weight were observed between p55^+/+^LDLR^−/−^ and p55^−/−^LDLR^−/−^ mice infused with AngII, while plasma cholesterol levels were approximately 25% reduced at the end of the study in p55^−/−^LDLR^−/−^ mice (Fig. 5A, B, C). To determine the effect of AngII infusion on plasma concentrations of inflammatory cytokines and chemokines, we performed a multi-cytokine analysis on plasma isolated at the end of the experiment. Comparable to our previous observations [10], plasma levels of MCP-1 were significantly and approximately 30% reduced in p55^−/−^LDLR^−/−^ mice, indicating that p55 TNFR signaling is important for regulating levels of MCP-1 in the circulation. Plasma levels of other chemokines or cytokines did not show significant differences between the groups (Fig. 5D).

### p55 TNFR signaling does not significantly affect AngII-induced AAA formation

**Figure 6:**
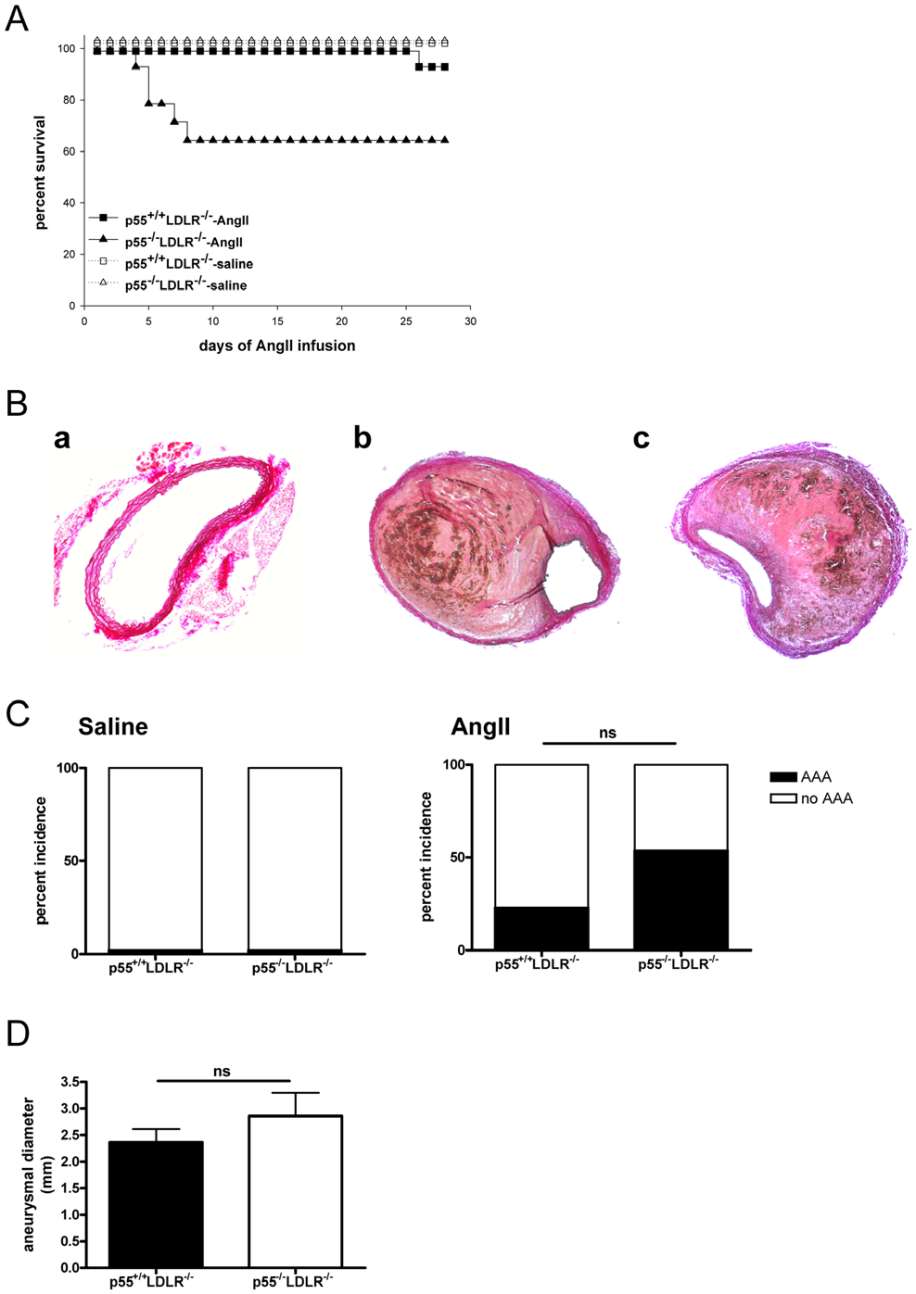
Survival curves, aneurismal incidence and suprarenal aortic diameters. (A) Percent survival in p55^+/+^LDLR^−/−^ (squares) and p55^−/−^LDLR^−/−^ (triangles) mice following infusion with saline (n = 3 mice/group) or AngII (n = 13–14 mice/group). Comparison of survival curves between AngII-infused p55^+/+^LDLR^−/−^ and p55^−/−^LDLR^−/−^ mice gave a probability value of p = 0.054 by Pearson's chi-square test. (B) representative cross-sections of EVG stained suprarenal aortas from (a) saline infused and of advanced AAA in (b) p55^+/+^LDLR^−/−^ and (c) p55^−/−^LDLR^−/−^ mice indicating dissecting aneurysms and formation of thrombi. Original magnification×100 (a) and×50 (b,c). (C) Percent incidence of AAA in p55^+/+^LDLR^−/−^ and p55^−/−^LDLR^−/−^ mice (p = 0.2 by Fisher's exact test). (D) Suprarenal aortic diameters in p55^+/+^LDLR^−/−^ (n = 12) and p55^−/−^LDLR^−/−^ (n = 8) mice (p = 0.3 by Student's t-test).

Neither genotype of saline-infused mice developed AAA. AngII infusion resulted in lethality due to AAA rupture in 5 out of 13 p55^−/−^LDLR^−/−^ within the first 10 days, while only one mouse out of 14 from the p55^+/+^LDLR^−/−^ group died due to AAA rupture, 26 days after the start of AngII administration. One additional mouse from the p55^+/+^LDLR^−/−^ group died due to ruptured thoracic aortic aneurysm (TAA) but presented no AAA and thus was not included in the lethality caused by ruptured abdominal aneurysms (Fig. 6A; p = 0.054). At the end of the infusion period (28 days), mice were sacrificed and the abdominal aortas were isolated and analysed. Total AAA incidence, quantified based on death before the end of the experiment due to a dissecting abdominal aortic aneurysm with presence of a retroperitoneal hematoma or, in the surviving mice, by the presence of a dissecting aneurysm in the suprarenal aorta with concomitant formation of a thrombus (Fig. 6B; b,c), was 23% in the p55^+/+^LDLR^−/−^ group (3 mice out of 13) compared to 54% in the p55^−/−^LDLR^−/−^ group (7 mice out of 13) (Fig. 6C; p = 0.22). In addition, measurement of the suprarenal aortic diameters in the mice that survived until the end of the experiment was not significantly different in the p55^−/−^LDLR^−/−^ mice compared to controls (Fig. 6D; p = 0.3).

Taken together, these findings indicate that p55 TNFR signaling does not appear to play a major modulating role in the formation of AngII-induced AAA.

## Discussion

Our results presented here indicate that p55 TNFR seems to play differential roles in the development of two vascular disorders, promoting atherosclerosis while not importantly affecting the formation of AAA induced by AngII. LDLR deficient mice lacking p55 TNFR showed reduced atherosclerotic lesion size in the aortic root. Moreover, accumulation of macrophages in the aortic arches, as assessed by CD68 expression, was reduced in p55 TNFR deficient mice at this site as well. The decrease in macrophage accumulation and atherosclerosis was concomitant with a reduced expression of adhesion molecules by the p55 TNFR knock-out endothelium covering atherosclerosis prone sites and a reduced expression of several pro-inflammatory and pro-atherosclerotic cytokines and chemokines. However, p55 TNFR deficiency did not significantly affect susceptibility to the development of AngII-induced abdominal aortic aneurysms.

We have previously shown that absence of p55 TNFR on hematopoietic cells protects against atherosclerosis development [10]. In a bone marrow transplantation setting we could determine that p55 TNFR deficiency results in a reduced scavenger receptor class A dependent uptake of modified lipoproteins by macrophages, leading to smaller foam cells and subsequent smaller atherosclerotic lesions. Moreover we showed that p55 TNFR deficiency in hematopoietic cells results in lower plasma levels of the crucial pro-atherosclerotic chemokine MCP-1, identifying an additional potential mechanism leading to smaller lesions. In the current study we extended these investigations to LDLR^−/−^ animals lacking p55 TNFR in all their cells. Hereby, we show that p55 TNFR is important for the expression of adhesion molecules on endothelial cells in atherosclerosis prone sites. An important downstream mediator of p55 TNFR signaling is the transcription factor NF-κB. In another recent study, we have shown that NF-κB signaling in endothelial cells is imperative for the development of atherosclerosis [14]. Using different mouse models targeting NF-κB activation specifically in endothelial cells, we determined that inhibition of NF-κB resulted in repressed expression of adhesion molecules such as VCAM-1 and ICAM-1 by the vascular endothelium and impaired macrophage recruitment to atherosclerotic plaques. Consequently, atherosclerotic lesions were smaller and characterized by strongly reduced expression of inflammatory cytokines, such as TNF and IL-6 and also reduced expression of atherosclerosis associated chemokines, such as MCP-1 and RANTES.

Different upstream receptors and pathways are responsible for activating NF-κB in endothelial cells and previous studies have shown that TLR receptors are upregulated in endothelial cells in atherosclerosis prone sites and positively contribute to plaque development [22], [23], [24]. In the present study we show that the TNF-p55 TNFR pathway is also an important activator of NF-κB and inducer of adhesion molecules in endothelial cells during atherogenesis, since VCAM-1, ICAM-1, e-selectin and p-selectin were reduced in the absence of p55 TNFR. Concomitant with a reduced NF-κB activation in the aortic arches, as assessed by IκBα expression, we also found reduced expression of TNF, IL-6, MCP-1 and RANTES, all proven to be important in atherosclerosis [25], [26]. Since, these factors are also expressed by macrophages we cannot exclude that their reduced levels are a reflection of smaller atherosclerotic lesions, instead of being a direct result of absence of p55 TNFR signaling. Plasma levels of these inflammatory mediators were not affected by absence of p55 (Fig. 4C) and showed a similar pattern as in the AAA study (figure 5B).

Our data are in line with recent observations by Zhang et al [11]. They showed that in a model of carotid artery-to-carotid artery interposition grafting, p55 TNFR expression on vascular cells promotes atherosclerosis. Similar to us they found that in their model, absence of p55 TNFR resulted in reduced expression of MCP-1, VCAM-1 and ICAM-1. We now show that these mechanisms are also applicable in diet-induced hyperlipidemic model of this disease.

In contrast to an aggravating role for p55 TNFR in atherosclerosis, this receptor does not seem to play a major role in the AngII-induced model of abdominal aortic aneurysm development. We found a mild reduction in plasma cholesterol levels in the absence of p55 TNFR, only in our AAA study and not in the atherosclerosis study. It has been shown before that inflammatory mediators can affect lipid metabolism (reviewed by Khovidhunkit et al. [27]). Interestingly, there is a species difference with respect to cholesterol metabolism. While lipopolysaccharide (LPS) and several proinflammatory cytokines downregulate cholesterol synthesis in man, they upregulate cholesterol levels in rodents. For TNF this was confirmed in several studies [28], [29] while other studies that were performed to investigate the effects of TNF in atherosclerosis did not find major effects on lipid levels (reviewed by Kleemann et al. [30]). Therefore, the potential effects of cytokine signaling on cholesterol metabolism may be context and model dependent. The fact that Angiotensin infusion in AAA mediates at least part of its effect through promoting inflammation and the fact that a different diet containing more saturated fat was used for the AAA studies, may explain why cholesterol levels in the two disease models respond differently with respect to p55 TNFR deficiency. As shown by us before [10], we also found reduced plasma levels of MCP-1 in the AAA study. This may potentially affect macrophage recruitment to the aneurysm areas, both in the initial phase upon AngII infusion and at later stages in response to thrombus formation.

Interestingly, while atherosclerosis is generally considered to be a vascular disease associated with Th1 cytokines [25], AAA was found to be dominated by Th2 cytokines [31]. In line with these findings we found that our animals from the atherosclerosis study showed higher levels of three Th1 cytokines (i.e. TNF, IL-12 and IFNγ) as compared to mice from the aneurysm study, while three Th2 cytokines (i.e. IL-4, IL-10 and IL-13) were not different between the two experiments (data not shown). It has been shown that manipulation of the Th1/Th2 balance highly influences the development of AAA in experimental settings. Shimizu et al. showed that inhibition of the Th2 cytokine IL-4 reduced the development of aneurysms while genetic ablation of the receptor for the Th1 cytokine IFNγ actually worsened the disease [32]. Hereby, they demonstrated that Th1 cytokines can protect against aneurysms while Th2 cytokines can aggravate this disease. Confirming this protective role for Th1 cytokines, it was recently shown that absence of both IFNγ or its downstream chemokine CXCL10 also ameliorate aneurysm development [33]. Thus, it could be expected that signaling by the main receptor of another Th1 cytokine like TNF would also be protective against aneurysms. In our experimental setting of AngII-induced AAA, p55 TNFR signaling appeared to confer some degree of protection from lethality due to ruptured abdominal aneurysms in the p55^+/+^LDLR^−/−^ group. However this effect was only marginal since differences in survival curves did not reach statistical significance and additional parameters of disease susceptibility like aneurismal incidence and average aortic diameters were also not significantly different between the groups. These data suggest that p55 TNFR has neither a clear protective nor an aggravating role in the development of AngII induced AAA. However, these mechanisms may only apply to this specific model of AngII-induced abdominal aortic aneurysm formation. It is for example known that anti-TNF works beneficial in animal models for Kawasaki disease associated coronary aneurysms [34]. Moreover, TNF antagonists are an important treatment for Kawasaki patients also suffering from acute vasculitis and coronary artery aneurysms [35].

Concluding, inhibition of p55 TNFR remains an attractive candidate for treatment of atherosclerosis since, in addition to our recently described pro-atherosclerotic function of p55 TNFR in regulating foam cell formation and systemic MCP-1 production [10], we here show that this receptor promotes atherosclerosis by inducing endothelial adhesion molecule expression and production of pro-atherosclerotic cytokines and chemokines at the arterial wall.

## Materials and Methods

### Ethics statement

All experiments were approved by the Committee for Animal Welfare of Maastricht University. The investigation conforms to the *Guide for the Care and Use of Laboratory Animals* published by the US National Institutes of Health (NIH Publication No. 85–23, revised 1996).

### Mice and diet

p55 TNFR knock-out mice (p55 TNFR^−/−^) on a C57BL/6 background were obtained from the Jackson Laboratory (Bar Harbor, ME) and have been described elsewhere [36]. Low-density lipoprotein (LDL) receptor knock-out mice (LDLR^−/−^) have been described elsewhere [37] and were crossed to C57BL/6 background for at least 10 generations. p55 TNFR^−/−^ mice were crossed to LDLR^−/−^ background to obtain double negative p55 TNFR^−/−^LDLR^−/−^ or littermate p55 TNFR^+/+^LDLR^−/−^ controls. All mice used were males. For the atherosclerosis study, mice (10–13 weeks of age) were fed a diet containing 16% fat, 0.15% cholesterol and no cholate (Hope Farms, Woerden, The Netherlands). For the aneurysm study, mice (8–12 weeks of age) were fed a diet supplemented with saturated fat (milk fat 21%) and cholesterol (0.15%; Harlan Teklad, diet # TD.88137). One week after initiation of high fat feeding, saline or AngII (1000 ng/kg/min) (Sigma A9525) was administered subcutaneously via Alzet osmotic minipumps (Model 2004; Durect Corporation, Cupertino, California, USA) for 28 days to induce suprarenal (dissecting) aneurysms as described previously [15].

### Genotyping and blood analysis

Primers for the wild-type or p55 TNFR deleted alleles were: 5′-TGTGAAAAGGGCACCTTTACGGC-3′; 5′-GGCTGCAGTCCACGCACTGG-3′; 5′-ATTCGCCAATGACAAGACGCTGG-3′, and for the wild-type or LDLR deleted alleles were: 5′-CATATGCATCCCCAGTCTTTG-3′; 5′-ATAGATTCGCCCTTGTGTCC-3′; 5′-GCAGTGCTCCTCATCTGACTTG-3′. Cholesterol and triglyceride levels were determined on plasma using enzymatic kits (Sigma-Aldrich; cat.no. 401 and 337). Plasma cytokine levels in AngII infused mice were determined with the Bio-Plex Mouse Cytokine 23-Plex Panel (cat.no:171-F11241; Bio-Rad) according to manufacturer's instructions.

### Atherosclerotic lesion analysis

Consecutive 7 µm sections of the heart the level of the atrioventricular valves were collected and were stained with toluidine blue for morphometric analysis and atherosclerosis quantification as previously described [14]. For VCAM-1 staining, sections were stained with anti-VCAM-1 antibody (BD).

### Quantitative gene expression

RNA was isolated from aortic arches using RNeasy columns with additional DNase digestion (Qiagen). cDNA synthesis was performed using the iScript™ cDNA synthesis kit (Bio-Rad) and quantitative PCR was performed using the qPCR iQ™ Custom SYBR Green Supermic (Bio-Rad) on an iCycler thermal cycler (Bio-Rad). Cyclophilin A was used to normalize RNA quantity. Primer sequences are available upon request.

### Quantification of aneurysms and morphometric analysis

For morphometric analysis of the AAA, the entire arterial tree was perfused with 10 ml PBS containing 0.1 mg/ml sodium nitroprusside (Sigma) following with 10 ml 1% paraformaldehyde (Sigma). The arterial tree was isolated and fixed overnight in 1% paraformaldehyde and embedded in paraffin. Cross sections (4 µm) of the suprarenal area of the abdominal aorta were made at 12 different levels at 200 µm intervals. Cross sections from each level were stained with H&E and elastic-Van Gieson (EVG). Total vessel area was quantified using the Leica Qwin V3.2.1 system and DM 500B microscope.

On necropsies of unexpected deaths, death due to rupture of aneurysm was qualified by presence of retroperitoneal hematoma in addition to AAA. Aneurysmal incidence was quantified based on such described deaths or, in the mice that survived until the end of the experiment, in the presence of a dissecting aneurysm in the suprarenal aorta with the concomitant formation of a thrombus. All analyses were performed without prior knowledge of the genotype.

### Statistical analysis

Statistical analyses were performed using Graphpad Prism (Graphpad Software) or SigmaPlot t-tests. For lesion area and characterization, differences were evaluated using a Welch corrected t-test. For comparison of survival curves the Pearson's chi-square test was used. Percent incidence of AAA was analyzed by Fisher's exact test. Data are expressed as means±SEM. A p value of less than 0.05 is considered statistically significant.
